# Editorial: 15 years of frontiers in neuroanatomy: the origin of Parkinson's disease

**DOI:** 10.3389/fnana.2025.1649700

**Published:** 2025-07-01

**Authors:** Barbara Falquetto, Cristina Nombela, Luiz R. G. Britto

**Affiliations:** ^1^Department of Pharmacology, Institute of Biomedical Sciences, University of São Paulo, São Paulo, Brazil; ^2^Biological and Health Psychology, School of Psychology, Universidad Autónoma de Madrid, Madrid, Spain; ^3^Department of Physiology and Biophysics, Institute of Biomedical Sciences, University of São Paulo, São Paulo, Brazil

**Keywords:** neurodegeneration, neuroprotection, Parkinson's disease, cognitive deficits, neuroinflammation

The original idea of the present Research Topic was to discuss the origins of Parkinson's disease, a topic that proved to still represent a huge challenge. With this in mind, we actually joined some experts in the disease and produced some articles on possible new frontiers for Parkinson's disease understanding and control. We pave here the way for new approaches for the characterization, diagnosis and management of that condition, recognizing this focus as more critical for parkinsonian patients at the present stage than the understanding of the exact origin of the disease.

For decades, PD was conceptualized primarily as a motor disorder, linked to the degeneration of dopaminergic neurons in the substantia nigra pars compacta. Over the past 20 years, however, a network-based view has emerged, recognizing that motor, cognitive, and affective symptoms stem from progressive dysfunction across interconnected neuroanatomical circuits. Cognitive deficits—particularly in executive control, divided attention, and visuospatial processing—often appear early and reflect disruption of the dorsolateral fronto-striatal circuitry, anterior cingulate cortex, and posterior parietal cortex, in parallel with the rostral-caudal spread of alpha-synuclein aggregates into limbic and cortical structures (Braak et al., 2003; Kehagia et al., 2010). The formal inclusion of diagnostic criteria for mild cognitive impairment in PD marked a major diagnostic shift (Litvan et al., 2012). Today, cognitive impairment is understood as a core and dynamic component of the parkinsonian syndrome, functionally and anatomically integrated into the broader neurodegenerative process, rather than an isolated or secondary domain.

In the above context, the study by Andújar-Castillo et al. provides empirical support for a domain-based approach to cognitive profiling in Parkinson's disease. Analyzing neuropsychological data from 316 patients across three centers, the authors identified three cognitive phenotypes—fronto-striatal, posterior cortical, and preserved—through latent cluster analysis. This framework, grounded in Movement Disorder Society criteria, reveals distinct trajectories of neurodegeneration and offers a standardized method for capturing cognitive heterogeneity in the disease, thereby enhancing cross-cohort comparability and informing more precise clinical interventions.

Brandão et al., also with a focus on cognitive aspects of Parkinson's disease, evaluated neuroanatomical aspects, such as cortical volume and thickness, in Parkinson's disease patients with or without mild cognitive impairment. Magnetic resonance imaging revealed that neuroanatomical changes across extensive fronto-temporo-parietal areas are correlated with cognitive performance, suggesting that those structural parameters could be used as markers of cognitive decline in Parkinson's disease.

With the general idea of focusing on the progression of the disease, Bianchetti et al. described the beneficial effects of a novel neuroinflammation inhibitor, AD-16 (also known as GIBH-130; Zhou et al., 2016), in the context of Parkinson's disease. Chronic inflammation, a hallmark of Parkinson's and other neurodegenerative diseases, plays a key role in disease development and progression. The authors observed neuroprotection, reduced microglia reactivity, and decreased levels of pro-inflammatory cytokines in a mouse model of the disease treated with the compound. AD-16 thus emerges as a promising therapeutic candidate for Parkinson's disease and other inflammation-related neurodegenerative conditions, especially in light of promising initial tests in humans (Peng et al., 2023).

Also with a focus on disease progression, Pedrão et al. reviewed here the pathological apoptosis as a key player in the progression of Parkinson's disease. Over the years, various cellular insults and phenotypes have been shown to trigger distinct death signaling events in affected neurons. This review offers a comprehensive overview of the main apoptotic pathways involved in the disease—intrinsic (mitochondrial) and extrinsic (inflammatory/oxidative stress)—and highlights recent advances in identifying potential therapeutic targets.

In conclusion, the present Research Topic adds new data on both the cognitive aspects of the disease in humans and neuroinflammation, cell death and disease progression in animal models. We feel that these complementary approaches represent an important avenue to advance the knowledge of Parkinson's disease pathophysiology, in the search of more effective markers of non-motor symptoms, in the understanding of the neuroanatomical changes and in relation to potential therapeutical targets. We are still in urgent need for those tools after more than 200 years of the initial description of the disease.
