# Correction: Editorial: 15 years of frontiers in neuroanatomy: the origin of Parkinson's disease

**DOI:** 10.3389/fnana.2025.1666562

**Published:** 2025-07-28

**Authors:** Barbara Falquetto, Cristina Nombela, Luiz R. G. Britto

**Affiliations:** ^1^Department of Pharmacology, Institute of Biomedical Sciences, University of São Paulo, São Paulo, Brazil; ^2^Biological and Health Psychology, School of Psychology, Universidad Autónoma de Madrid, Madrid, Spain; ^3^Department of Physiology and Biophysics, Institute of Biomedical Sciences, University of São Paulo, São Paulo, Brazil

**Keywords:** neurodegeneration, neuroprotection, Parkinson's disease, cognitive deficits, neuroinflammation

In the published article, the funding sources that supported this publication were erroneously omitted. The corrected Funding statement appears below.

